# Preventive and Therapeutic Roles of Berberine in Gastrointestinal Cancers

**DOI:** 10.1155/2019/6831520

**Published:** 2019-12-28

**Authors:** Siwang Hu, Ruochi Zhao, Yahui Liu, Junzheng Chen, Zhijian Zheng, Shuangshuang Wang

**Affiliations:** ^1^Department of Spine Surgery, The Affiliated Wenling Hospital of Wenzhou Medical University, Wenling 317500, China; ^2^Department of Cardiology, Ningbo First Hospital, Ningbo 315000, China; ^3^Central Laboratory, Ningbo First Hospital, Ningbo 315010, China; ^4^Department of General Surgery, The Affiliated Wenling Hospital of Wenzhou Medical University, Wenling 317500, China

## Abstract

Berberine (BBR) is an isoquinoline alkaloid isolated from various types of plants, including those from the Berberidaceae, Ranunculaceae, and Papaveraceae families. It has long been used in traditional Chinese medicine for treating diarrhea and gastrointestinal disorders. The medicinal properties of BBR include antimicrobial, anti-inflammatory, antioxidative, lipid-regulatory, and antidiabetic actions. Importantly, the efficacy of BBR against cancers has been assessed in several experimental studies and clinical trials. Gastrointestinal (GI) cancers are a group of the most prevalent cancers worldwide that are associated with high morbidity and mortality, and their associated mortality has been increasing over the years. Thus, GI cancers have become a burden to the patients and health care systems. This review summarizes the cellular and molecular mechanisms underlying the therapeutic effects of BBR and explores its potential preventive and therapeutic applications against GI cancers.

## 1. Introduction

### 1.1. Sources and Pharmacological Effects of Berberine (BBR)

**Figure 1:**
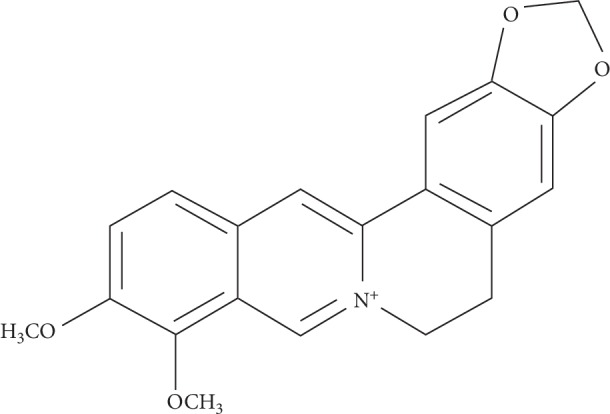
Chemical structure of berberine.

BBR is a benzyl tetra isoquinoline alkaloid (2,3-methylenedioxy-9,10-dimethoxyprotoberberine chloride; C_20_H_18_NO_4_^+^) with a molar mass of 336.36122 g/mol (Figure 1). It is a well-known phytochemical compound extracted from the roots of various plants, such as *Berberis vulgaris*, *B. aristata*, *B. aquifolium*, *Hydrastis canadensis*, and *Phellodendron chinense*, and Coptidis rhizoma, i.e., the dried rhizome of *Coptis japonica* and *C. chinensis* [1, 2].

BBR-containing plants have been used medicinally for at least 3000 years in many traditional medicine systems, including ancient Chinese, Egyptian, Ayurvedic, and Iranian medicine. In traditional Chinese medicine, BBR is generally administered to patients with gastrointestinal (GI) disorders, especially gastroenteritis. In recent years, BBR has attracted considerable attention because of its diverse pharmacological properties, low toxicity, and low cost. Several pharmacological properties of BBR have recently been identified, including antimicrobial, anti-inflammatory, antioxidant, antidiabetic, lipid-regulatory, sedative, antiemetic, antinociceptive, and anticholinergic effects [3–5]. Furthermore, many studies have shown that BBR can be used for treating hypertension, cardiovascular diseases (due to antiheart failure, antiarrhythmia, and antiplatelet aggregation effects), neuronal diseases, gastrointestinal disorders, and several types of cancers [6–10]. The molecular and cellular mechanisms underlying the therapeutic effects of BBR, such as anti-inflammatory, antiapoptotic, antioxidative, and autophagy-promoting activities, have been found to involve some signaling pathways, such as the mitogen-activated protein kinase (MAPK) signaling, phosphatidylinositol-3 kinase/AKT/mammalian target of rapamycin (PI3K/Akt/mTOR), the Janus Kinase 2/signal transducer and activator of transcription 3 (JAK2/STAT3), and the nuclear factor erythroid 2-related factor 2/hemeoxygenase-1 (Nrf2/HO-1) pathways [11].

Because of its low water solubility, the oral bioavailability of BBR is poor; less than 5% of orally administered BBR gets absorbed through the intestinal wall. Intestinal P-glycoprotein, an important transporter protein located in the epithelial cell membrane, contributes to this poor bioavailability by functioning as an efflux pump to actively expel the alkaloid outside the luminal mucosal cells. Thus, the administration of P-glycoprotein inhibitors to enhance BBR absorption is a potential strategy to improve BBR bioavailability. The administration of BBR in its absorbable form dihydroberberine (dhBBR) can also improve its bioavailability. Essentially, BBR is converted into dhBBR via reduction by the nitroreductases of gut microbiota, whereas dhBBR is reverted to BBR via nonenzymatic oxidation in the intestine. Therefore, theoretically, the coadministration of probiotics (to regulate gut microbiota) with BBR could be useful in improving BBR bioavailability.

### 1.2. BBR in Cancer Treatment

The most common cancer treatment strategies include surgical resection, radiotherapy, and chemotherapy. In recent years, treatment strategies such as targeted therapy and immunotherapy have introduced significant breakthroughs in cancer therapy. Moreover, during the last decade, several clinical trials and laboratory experiments have been conducted to ascertain BBR's efficacy in treating cancer. In these studies, BBR has demonstrated anticancer activities against the proliferation, growth, angiogenesis, and metastasis of a variety of tumors, including oral cancer, esophageal cancer, pancreatic cancer, gastric carcinoma, colorectal cancer, colon cancer, liver cancer, lung cancer, nasopharyngeal carcinoma, breast cancer, endometrial cancer, cervical cancer, ovarian cancer, bladder cancer, prostate cancer, and melanoma.

## 2. Epidemiology of GI Cancers

GI cancers, including esophageal, gastric, pancreatic, liver/bile duct, small bowel, and colorectal cancers, are the most widespread malignancies worldwide. Globally, of the 14 million people diagnosed with cancer each year, 4 million have GI cancers. Thus, the incidence of GI cancers is greater than that of lung and breast cancers combined. In addition, roughly half of all cancer-related deaths are attributable to GI cancers [12, 13], indicating that GI cancers are the leading cause of cancer-related mortality. The data from Surveillance, Epidemiology, and End Results revealed that in 2016, GI cancers accounted for approximately 16.9% of the 160,000 newly diagnosed cancer cases, and 24.2% of all cancer-related deaths in the USA. According to China cancer statistics from 2018, the top five common cancers in China are lung cancer (24.63%), gastric cancer (13.62%), liver cancer (12.72%), colorectal cancer (10.13%), and esophageal cancer (8.77%). Thus, GI cancers accounted for nearly 50% of the cancer cases, and their incidence is increasing every year.

In this review, we summarize the pharmacological effects and potential cellular and molecular targets of BBR in GI cancer with a view to expanding its clinical applications.

## 3. BBR in GI Cancers

### 3.1. Esophageal Cancer

In 2000, Iizuka investigated the anticancer and anticachectic effects of Coptidis rhizoma (CR) in nude mice with esophageal tumors that constitutively secreted interleukin-6 (IL-6) and caused cachexia. The author found that orally administered CR did not affect the proliferation of esophageal cancer cells but prevented the weight loss of tumor-bearing mice and reduced tumor and serum IL-6 levels. The results also confirmed that BBR dose-dependently repressed the mRNA expression and secretion of IL-6 in esophageal cancer cells in vitro [14]. Jiang et al. found that BBR dose- and time-dependently suppressed the proliferation of and enhanced the apoptosis of KYSE-70 cells, a human esophageal squamous carcinoma cell line. They also found that BBR enhanced the cell cycle arrest of KYSE-70 cells in the G2/M phase by elevating the expression of the cell cycle protein p21. Further, BBR significantly retarded the phosphorylation of Akt and the expression of the mammalian target of rapamycin (mTOR), and its downstream target p70S6K and continuously promoted the phosphorylation of AMP-activated protein kinase (AMPK). Thus, the Akt/mTOR pathway, which plays a vital role in controlling cell growth and apoptosis, and its negative regulator AMPK may be involved in BBR's anticancer function [11]. Mishan et al. found that BBR dose-dependently retarded the proliferation and invasion of esophageal cancer cells by downregulating the expression of CXC chemokine receptors 4 and 7, which are known to promote tumor proliferation and invasion [15]. Ren et al. reported that the combination of BBR and galangin shows potent synergistic anticancer activity in esophageal cancer both in vitro and in vivo. In particular, the combination was shown to inhibit cancer cell growth, increase cancer cell apoptosis, and cell cycle arrest in the G2/M phase and upregulate the levels of reactive oxygen species (ROS) in cancer cells. Treatment with BBR alone downregulated the Wnt3a and *β*-catenin expression required for the Wnt/*β*-catenin pathway, which plays a major role in initiating cancer progression. Moreover, the BBR and galangin combination not only suppressed Wnt3a and *β*-catenin expression but also induced apoptosis in esophageal cancer cells [16].

Radiotherapy is an important treatment method for esophageal squamous cell carcinoma (ESCC). Yang et al. found that the radiosensitivity of xenografts in nude mice and ESCC cells was significantly enhanced by BBR via the suppression of vascular endothelial growth factor (VEGF) and hypoxia inducible factor-1 alpha (HIF-1*α*) expression [17]. Liu et al. showed that increased levels of the homologous recombination repair protein RAD51 enhance resistance against radiotherapy and chemotherapy. In their study, the downregulation of RAD51 expression using RNA interference improved the radiosensitivity of cancer cells. These findings prove that RAD51 plays a fundamental role in regulating radiosensitivity. The authors also found that BBR pretreatment significantly decreased RAD51 levels in ESCC cells, thereby increasing their radiosensitivity. However, the introduction of exogenous RAD51 may significantly counteract the radiosensitizing effect of BBR [18].

### 3.2. Gastric Cancer (GC)

Overexpression of matrix metalloproteinases (MMPs) may participate in the invasion and metastasis of malignant cells by degrading all extracellular matrix components. Four MMPs (MMP-1, -2, -7, and -9) have been confirmed to be associated with GC [19–21]. Lin et al. reported that BBR dose-dependently suppresses the growth and migration of human GC cells. Their results showed that BBR increased ROS production and reduced NF-*κ*B and p65 levels in GC cells. BBR was also found to inhibit the mRNA expression of and suppress the protein levels of MMP-1, -2, and -9 [22].

Akt plays crucial regulatory roles in different cellular processes, including survival, proliferation, metabolism, differentiation, and apoptosis [23]. It is highly expressed and constitutively activated in GC cells. Yi et al. found that BBR treatment suppresses GC cell growth both in vitro and in vivo. Their results showed that BBR-induced human GC cell apoptosis in vitro by upregulating the expression of caspase-3 and cleaved poly(ADP-ribose) polymerase and disturbing the mitochondrial membrane potential. In addition, BBR repressed the activation of Akt and the Akt/mTOR/p70S6/S6 signaling pathways in human GC cells. BBR was also found to suppress GC growth in xenograft nude mice models. These findings suggest that the Akt pathway plays a key role in BBR-induced GC cell apoptosis, which might be a pivotal molecular mechanism underlying the anticancer effects of BBR [24].

Survivin, an apoptosis inhibitor, has been discovered to play a vital role in preventing the initiation and progression of apoptosis and accelerating cell cycle progression [25, 26]. Signal transducer and activator of transcription 3 (STAT3) is a crucial transcription factor that enhances the expression of survivin and has been shown to be constitutively activated in early-stage GC [27]. Survivin and STAT3 signaling have been proven to be crucial determinants of chemoresistance in GC. Pandey found that BBR dose-dependently inhibited GC cell viability by suppressing STAT3 levels and survivin expression. Notably, 5-fluorouracil in combination with BBR enhanced gastric adenocarcinoma cell death by downregulating survivin and STAT3 expression. These findings suggest that BBR can be used as an adjunct therapy to alleviate chemoresistance during GC treatment [28].

### 3.3. Colorectal Cancer (CRC)

Upregulation of the expression of cyclooxygenase-2 (COX-2) and its main downstream product prostaglandin E2 (PGE2) strongly promotes CRC cell proliferation, attachment, migration, and tumor angiogenesis and inhibits cancer cell apoptosis. Constitutive activation of the JAK2/STAT3 signaling pathway in CRC upregulates the expression of its downstream genes, such as MMP-2/-9, thereby enhancing the migration and metastasis of cancer cells. Liu found that BBR represses the proliferation and migration of CRC cells in vitro and in vivo by inhibiting JAK2 and STAT3 phosphorylation, which prevents the increase in COX-2/PGE2 levels and consequently decreases MMP-2/-9 expression [29]. Cai et al. found that BBR time- and dose-dependently inhibits the proliferation of human colorectal adenocarcinoma cells by increasing cell cycle arrest in the G2/M phase. They also reported that the BBR-induced suppression of colorectal adenocarcinoma cell growth is associated with the downregulation of cell cycle proteins, including cyclin B1, cdc2, and cdc25c [30]. Li et al. found that BBR inhibits colon tumorigenesis in the colorectal carcinogenesis mouse model by activating AMPK, a key regulator of cell metabolism, and inhibiting its downstream target mTOR. Their in vitro experiment showed that BBR exerts antiproliferative and proapoptotic effects on CRC cells via AMPK-dependent inhibition of the mTOR signaling pathway and prevents NF-*κ*B activation, decreases cyclin D1 and survivin levels, induces p53 phosphorylation, and enhances caspase-3 cleavage in an AMPK-independent manner [31]. Nonsteroidal anti-inflammatory drug-activated gene (NAG-1) and activating transcription factor 3 (ATF3) possess proapoptotic and antitumorigenic activities. Piyanuch et al. found that BBR inhibits the proliferation and induces the apoptosis of human CRC cells by increasing ATF3 levels in a p53-dependent manner and upregulating NAG-1 expression via multiple signaling pathways [32]. Li et al. showed that BBR inhibited colonic epithelium hyperproliferation and colitis-associated tumorigenesis in colitis-associated colorectal cancer (CACRC) mice model by inhibiting tumor necrosis factor-*α* (TNF-*α*) and IL-6 expression in colonic macrophages. EGFR–ERK signaling downstream of the BBR/proinflammatory cytokine axis plays a crucial role in reducing CACRC cell proliferation [33]. La found that BBR can enhance stress-induced autophagy of colon cancer cells by upregulating the expression of glucose regulated protein 78 (GRP78), which is achieved by the binding of BBR to GRP78 to form a complex that evades ubiquitination and proteasomal degradation [34]. Hyperactivated glucose uptake and glycolytic metabolism are regarded as a hallmark of cancer. Hypoxia inducible factor one alpha (HIF-1*α*), a well-known transcription factor, is known to regulate glucose metabolism in cancer cells. Mao et al. found that BBR inhibits the growth of colon cancer cells and glucose uptake and the transcription of glucose metabolic genes (GLUT1, LDHA, and HK2) by repressing HIF-1*α* expression in an mTOR-dependent manner [35].

In a study by Zhang, BBR was found to reduce the number and size of intestinal polyps in ApcMin/+ mice, in addition to reducing the Wnt activity and downregulating the expression of its target genes, cyclin D1 and c-Myc. In a clinical trial, oral administration of BBR was also found to diminish the polyp size and reduce cyclin D1 expression in the polyp samples of patients with familial adenomatous polyposis patients [36]. Consistent with these findings, Ruan et al. reported that BBR suppressed the growth of intestinal polyp by inhibiting the *β*-catenin signaling pathway in animals and patients with familial adenomatous polyposis. They found that BBR directly bound to the nuclear receptor retinoid X receptor alpha (RXR*α*) at the region containing Gln275, Arg316, and Arg371 residues and promoted its interaction with nuclear *β*-catenin, leading to c-Cbl-mediated degradation of *β*-catenin and consequent prevention of colon cancer cell proliferation. In addition, BBR inhibited the development of human colon carcinoma xenograft in nude mice in an RXR*α*-dependent manner [37]. These findings indicate that BBR inhibits the formation of colon tumors from familial adenomatous polyposis.

### 3.4. Pancreatic Cancer

Cancer stem cells play an important role in metastasis and the relapse of drug-resistant cancers. Side population (SP) cells have the ability to exclude Hoechst 33342 dye and are referred to as cancer stem cells. Park et al. investigated the effect of BBR on pancreatic cancer stem cells and found that BBR decreased SP and the expression of stem cell-associated genes (SOX2, POU5F1, and NANOG) in the pancreatic cancer cell lines PANC-1 and MIA PaCa-2 [38]. In their next study, they found that BBR inhibited the proliferation of these cell lines in a dose-dependent manner by inducing cell cycle arrest in the G1 phase and apoptosis. Notably, at half-maximal inhibitory concentration, BBR's apoptotic effect on pancreatic cancer cells was greater via ROS generation than via caspase 3/7 activation [39]. Pinto-Garcia et al. reported that BBR exhibits preferential cytotoxicity toward pancreatic cancer cells over normal cells. Its cytotoxicity on pancreatic cancer cells was found to be accompanied by the activation of BRCA1-mediated DNA damage response, G1/S and G2/M cell cycle checkpoint regulation, and p53 signaling pathway [40].

### 3.5. Cholangiocarcinoma and Hepatocellular Carcinoma (HCC)

Many studies have demonstrated that the therapeutic effect of BBR on cholangiocarcinoma and HCC occurs via inhibiting cancer cell proliferation and promoting cancer cell apoptosis. Puthdee et al. demonstrated that the inhibitory effects of BBR on the proliferation of hamster cholangiocarcinoma cells in vitro and in vivo occurred via the induction of cell cycle arrest in the G1 phase [41]. Li et al. reported that BBR induces the cell cycle arrest of HCC cells in the G0/G1 phase by enhancing CDKIs p21Cip1 and p27Kip1 expression via Akt/FoxO3a/Skp2 axis regulation [42]. Saxena et al. showed that BBR induces mitochondrial impairment and apoptosis of human hepatoma cells by modulating the PHLPP2-Akt-MST1 kinase signaling pathway [43].

Huang et al. observed that the combination of BBR and sorafenib synergistically inhibited the proliferation of HCC cells and induced apoptosis in a dose- and time-dependent manner by increasing the expression of cleaved poly(ADP-ribose) polymerase and cleaved caspase-3 and decreasing that of the antiapoptotic proteins BCL-2 and VEGF [44]. Dai et al. found that BBR combined with HMQ1611 (BCH) exerted a strong synergistic effect on HCC both in vitro and in vivo, and the potential mechanism underlying this effect involved the inhibition of the Wnt/*β*-catenin signaling pathway [45].

## 4. Noncoding RNA Regulation

Human genome sequencing has revealed that more than 80% of the human genome is transcribed to a versatile group of RNA transcripts called noncoding RNAs (ncRNAs), which, as the name suggests, do not encode proteins. Based on their nucleotide (nt) length, they are classified into three types: <50-nt ncRNAs, including microRNAs (miRNAs), small interfering RNAs (siRNAs), and piwi-interacting RNAs (piRNAs); 50–500-nt ncRNAs, including rRNAs, tRNAs, small nuclear RNAs (snRNAs), small nucleolar RNAs (snoRNAs), long ncRNAs (lncRNAs), SLRNAs, and SRPRNAs; and >500-nt ncRNAs, including mRNA-like ncRNAs. Among them, miRNAs and lncRNAs have been extensively examined over the past two decades and have been found to be involved in the onset, development, and progression of several cancers. BBR has also been found to regulate the expression of miRNAs and lncRNAs in GI cancers.

### 4.1. MicroRNAs

MiRNAs are short 20–22-nt-long ncRNAs that repress the translation of their target mRNAs by binding to their 3′-untranslated region (UTR) by imperfect complementary matches. Abnormal levels of miRNAs have been found in various pathological states, such as infectious diseases, cardiovascular diseases, endocrine diseases, and cancers.

Impaired regulation of miRNAs has been observed throughout various stages of GI cancers, and targeting the dysregulated miRNAs has been demonstrated as a new treatment strategy for GI cancers. It has been shown that BBR can inhibit GI cancer development by regulating the expression of miRNAs associated with it.

#### 4.1.1. Gastric Cancer

Yang showed that BBR prevents the growth of SGC-7901 GC cells and enhances their cell cycle arrest in the G1 phase and apoptosis. Using RNA and miRNA sequencing technologies, 347 upregulated, 93 downregulated, and 78 novel miRNAs were identified, which were found to be involved in cell growth, metabolism, cell junction, inflammation, apoptosis, acetylation, and TGF-*β* and Wnt signaling pathways [46].

In recent studies, miRNA-203 has been considered as a neoplasm biomarker that inhibits carcinoma development [47, 48]. Chemoresistance is a major reason for the failure of GC treatment. You et al. found that BBR increases cisplatin sensitivity and induces apoptosis in cisplatin-resistant GC cells via a caspase-dependent pathway. In their study, BBR treatment increased the expression of miRNA-203, which directly targets the 3′-UTR of Bcl-w, a member of Bcl-2 family that plays a crucial role in accelerating the invasion and metastasis of GC cells by increasing MMP-2 level via PI3K, Akt, and Sp1 activation. In addition, miRNA-203 overexpression showed a cisplatin-sensitizing effect like that reported for BBR. Taken together, these findings suggest that BBR reduces the cisplatin resistance of GC cells by modulating the miRNA-203/Bcl-w apoptotic signaling pathway [49].

#### 4.1.2. Colorectal Cancer

Huang et al. investigated the effects of BBR and evodiamine on the interaction between DNA methyltransferases (DNMTs) and their target miRNAs in a TGF-*β*1-induced CRC model. They found markedly decreased expression of DNMT1, DNMT3A, DNMT3B, and their target miRNAs (miRNA-152, miRNA-429, and miRNA-29a) in the colon cancer tissues after 24 h incubation with TGF-*β*1. Notably, both BBR and evodiamine increased the expression of DNMT1, DNMT3A, DNMT3B, and their target miRNAs. These findings suggest that BBR and evodiamine suppress the onset and development of colon cancer by regulating the expression of DNMTs and miRNAs [50].

Li et al. found that miRNA-429 levels are increased in human CRC tissues and that this increase is significantly associated with tumor size, lymph node invasion, and an unfavorable prognosis. In their study, overexpressed miRNA-429 inhibited cell apoptosis by directly targeting SOX2 in CRC cells, suggesting that miRNA-429 plays an oncogenic role in CRC and can thus be used as a new prognostic biomarker for CRC [51]. Liu et al. investigated the effect of BBR and evodiamine on CRC and miRNA-429 expression using an in vitro culture of colorectal tissue and found that BBR and evodiamine exert therapeutic effects on CRC by downregulating miRNA-429 expression [52].

Some recent studies have demonstrated the anticancer activity of miRNA-296 in various cancers, such as breast, lung, pancreatic, colorectal, colon, cervical, and prostate cancers. However, one study showed that progressive downregulation of miRNA-296 during tumor development is associated with cancer invasion and metastasis [53]. Pin1, a peptidyl-prolyl isomerase, regulates the expression of proteins essential for cell cycle progression, such as cyclin D1 [54]. Su et al. revealed that the combination of NVP-AUY922 (a Hsp90 inhibitor) and BBR retards human colon adenocarcinoma cell proliferation by inhibiting cyclin dependent kinase 4 (CDK4) expression and inducing miRNA-296-mediated suppression of the Pin1-*β*–catenin–cyclin D1 signaling pathway. These findings suggest that the therapeutic effects of the combination of NVP-AUY922 and BBR occur via the inhibition of multiple oncogenic signaling pathways [55].

#### 4.1.3. Hepatocellular Carcinoma

miRNA-122 is a tissue-specific miRNA that is most abundant in the liver and is involved in fatty acid and cholesterol metabolism and hepatitis C viral replication. Downregulation of miRNA-122 expression is frequently observed in HCC cells and has been shown to elevate *α*-fetoprotein levels and produce a more biologically aggressive HCC phenotype. Chai et al. found that coptisine inhibits the proliferation and migration and promotes the apoptosis of HCC cells both in vitro and in vivo. Coptisine was shown to inhibit tumor growth in nude mice via the upregulation of miR-122 expression [56].

### 4.2. Long Noncoding RNAs

LncRNAs are >200-nt-long ncRNAs that regulate the expression of target genes in various biological processes, such as chromatin modification, transcription, and posttranscription. Accumulating evidence has indicated that the aberrant expression of lncRNAs may induce the onset and progression of various cancers. Many studies have reported that BBR treatment can modulate lncRNA expression in various diseases, such as diabetes [57], poststroke inflammation [58], and nonalcoholic fatty liver disease [59]. However, evidence suggesting the involvement of lncRNAs in the anticancer effects of BBR is limited.

Dai et al. showed that CRC treatment with BBR dose-dependently represses cell proliferation and enhances cell apoptosis. In their functional experiment, BBR exerted its effects by inducing the overexpression of lncRNA cancer susceptibility candidate 2 (CASC2), the downstream target of which was found to be the antiapoptotic gene BCL2. Further, lncRNA CASC2 silenced BCL2 expression by binding to the promoter region of BCL2 in an EZH2-dependent manner. These results suggest that BBR functions as a CRC inhibitor by regulating the CASC2/EZH2/BCL2 axis [60].

## 5. Gut Microbiota

Gut microbiota are one of the vital constituents of the human body. Accumulating evidence has shown that gut microbiota are closely associated with the onset and progression of CRC through its interaction with the surrounding environment. Several studies have demonstrated that BBR accumulates in the intestine, regulates gut microbiota, and confers beneficial effects against insulin resistance, impaired lipid metabolism, atherosclerosis, and diabetes [61–63].

In the study by Wang, the microbiota imbalances occurred in Apc min/+ mice fed with a high-fat diet (HFD), while BBR mitigated the imbalances. Notably, *Verrucomicrobia,* which exhibit proinflammatory properties, was significantly upregulated in Apc min/+ mice fed with an HFD, while it was downregulated after BBR treatment. At the genus level, they found the abundance of the genus *Akkermansia*, belonging to the *phylum Verrucomicrobia*, was significantly increased in the HFD group. In addition, *Akkermansia* abundance was found to be enhanced in CRC patients. *Akkermansia* is known to be a mucin degrader that is associated with intestinal inflammation and has been reported to be strongly associated with the rate of tumorigenesis. In their study, BBR diminished intestinal cancer development and modified gut microbiota composition in high-fat diet-fed mice with multiple intestinal neoplasias. At the genus level, BBR decreased the abundance of *Akkermansia*, blocked mucin degradation, and increased that of some short chain fatty acid-producing bacteria. These findings suggest that BBR prevents the onset and development of CRC by modifying the gut microbiota composition [64].

Studies have reported that *Fusobacterium nucleatum* can accelerate the onset and development of intestinal tumors by modulating the tumor microenvironment [65]. In particular, Yu showed that the accumulation of *F. nucleatum* in the gut accelerated the onset of colonic tumors in mice. BBR treatment alleviated the *F. nucleatum*-mediated increase in opportunistic pathogens and decreased the secretion of IL-21/22/31 and CD40L and the expression of p-STAT3, p-STAT5, and p-ERK1/2 in vivo. This finding suggests that BBR suppresses *F. nucleatum*-induced CRC onset by modulating the tumor microenvironment and preventing the activation of tumorigenesis‐related pathways [66].

## 6. Conclusions

As shown in Table 1, BBR exerts several therapeutic effects on GI cancer cells via its involvement in various pathways, such as the cell cycle, inflammation, autophagy, and apoptosis. BBR also modulates the levels of various types of pro- and antitumor miRNAs and lncRNAs. As an adjuvant to chemotherapy or radiotherapy, BBR has been shown to enhance the therapeutic effects in GI cancers. Furthermore, BBR has been found to modulate the tumor immune microenvironment by regulating the gut microbiota composition. BBR has long been used as a medicinal agent in traditional medicine systems, and recent studies have proven that it could be used as a potent anticancer agent in the clinical treatment of GI cancers.

## Figures and Tables

**Table 1:** Suppressive effect of BBR on GI cancer.

Drugs	Type of regulation	Mechanism	Cell lines/animal model	References
Coptidis rhizome (CR)	Anticachectic	IL-6↓	Esophageal cancer model	Iizuka et al. [14]
BBR	Antiproliferation Proapoptosis	G2/M arrest↑ P21↑ Akt/mTOR↓ p-AMPK↑	Human esophageal squamous carcinoma cell line KYSE-70 cells	Jiang et al. [11]
BBR	Antiproliferation Anti-invasion	Cxc chemokine receptor 4 and Cxc chemokine receptor 7↓	Esophageal cancer cells	Mishan et al. [15]
BBR and galangin	Antiproliferation Proapoptosis	G2/M arrest↑ ROS↑Wnt3a ↓*β*-catenin↓	Esophageal cancer cells/model	Ren et al. [16]
BBR	Proradiosensitivity	VEGF and HIF-1*α*↓	Esophageal squamous cell carcinoma	Yang et al. [17]
BBR	Proradiosensitivity	RAD51↓	Esophageal cancer cells	Liu et al. [18]
BBR	Antiproliferation Antimigration	ROS↑NF-*κ*B p65↓MMP-1 -2, and -9↓	GC cells	Lin et al. [22]
BBR	Proapoptosis	Caspase-3↑ cleaved poly ADP-ribose polymerase↑ The damage of mitochondrial membrane potential Akt/mTOR/p70S6/S6↓	GC cells	Yi et al. [24]
BBR	Antiproliferation	STAT3↓survivin↓	Gastric adenocarcinoma cells	Pandey et al. [28]
BBR and 5-FU	Antichemotherapy resistance	STAT3↓survivin↓	Gastric adenocarcinoma cells	Pandey et al. [28]
BBR	Antiproliferation Antimigration	COX-2/PGE2↓ p-JAK2↓p-STAT3↓MMP-2/-9↓	CRC cells	Liu et al. [29]
BBR	Antiproliferation	G2/M arrest↑ cyclin B1↓cdc2↓cdc25c↓	Human colorectal adenocarcinoma cells	Cai et al. [30]
BBR	Antiproliferation Proapoptosis	AMPK↑mTOR↓NF-*κ*B↓cyclin D1↓survivin↓p-p53↑caspase-3↑	Colorectal carcinogenesis mouse model CRC cells	Li et al. [31]
BBR	Antiproliferation Proapoptosis	ATF3 ↑ NAG-1↑	CRC cells	Piyanuch et al. [32]
BBR	Antiproliferation Antitumorigenesis	TNF-*α*↓ IL-6↓in macrophages EGFR-ERK signaling↓	Colitis-associated colorectal cancer (CACRC) mice model and RAW 264.7 macrophages and colon cancer HCT116 cells	Li et al. [33]
BBR	Proautophagy	GRP78↑	Colon cancer cells	La et al. [34]
BBR	Antiglucose uptake Antiglucose metabolic	GLUT1↓LDHA↓HK2↓ HIF-1*α* protein↓ mTOR phosphorylation↓	Colon cancer cells	Mao et al. [35]
BBR	Anti-intestinal polyps	Wnt↓cyclin D1↓ c-Myc↓	ApcMin/+ mice	Zhang et al. [36]
BBR	Anti-intestinal polyps	*β*-Catenin signaling pathway↓	Nude mice	Ruan et al. [37]
BBR	Anticancer stem cells Antimetastasis	Cancer stem cells↓ SOX2↓, POU5F1↓, NANOG↓	Pancreatic cancer cells	Park et al. [38]
BBR	Antiproliferation Proapoptosis	G1/S arrest↑ ROS↑	Pancreatic cancer cells	Park et al. [39]
BBR	Antiproliferation Proapoptosis	Caspase-3↑caspase-7↑ BRCA1-mediated DNA damage↑ G1/S arrest ↑G2/M arrest↑ P53 signaling pathways ↑	Pancreatic cancer cells	Pinto-Garcia et al. [40]
BBR	Antiproliferation	G1/S arrest ↑	Hamster cholangiocarcinoma (CCA) cells	Puthdee et al. [41]
BBR	Antiproliferation	G0/G1 arrest ↑CDKIs p21Cip1↑ p27Kip1↑	HCC cells	Li et al. [42]
BBR	Promitochondrial impairment Proapoptosis	PHLPP2-Akt-MST1 kinase signaling pathway	HCC cells	Saxena et al. [43]
BBR and sorafenib	Antiproliferation Proapoptosis	ADP-ribose polymerase↑ caspase-3↑ BCL-2↓VEGF↓	HCC cells	Huang et al. [44]
BBR and HMQ1611 (BCH)	Antiproliferation Antimigration	G1 phase arrest ↑ Wnt/*β*-catenin signaling pathway↓	HCC cells	Dai et al. [45]
BBR	Antiproliferation Proapoptosis	347 miRNAs ↑ 93 miRNAs↓ 78 novel miRNAs were identified	GC cells	Yang et al. [46]
BBR	Cisplatin sensitivity↑ Proapoptosis	miRNA-203↑Bcl-w↓	GC cells	You et al. [49]
BBR	Antiproliferation Antitumorigenesis	DNMT1↑, DNMT3A↑, DNMT3B↑ and miRNA-152↑, miRNA-429↑, miRNA-29a↑	Colon cancer tissues	Huang et al. [50]
BBR	Proapoptosis	miRNA-429↓	Colorectal tissue	Liu et al. [52]
BBR and NVP-AUY922	Antiproliferation	CDK4↓miR-296↑Pin1-*β*-catenin-cyclin D1 signaling pathway↓	Human colon adenocarcinoma cells	Su et al. [55]
coptisine (COP)	Antiproliferation Antimigration Proapoptosis	miR-122↑	HCC cells hepatocellular carcinoma nude mice	Chai et al. [56]
BBR	Antiproliferation Proapoptosis	lncRNA CASC2↑BCL2↓	CRC cells	Dai et al. [60]
BBR	Antidevelopment of intestinal cancer modified gut microbiota's structure	Akkermansia↓ Short chain fat acid (SCFA)-producing bacteria↑	Multiple intestinal neoplasia mice	Wang et al. [64]
BBR	Antigenesis of CRC induced by *Fusobacterium nucleatum*	Fusobacterium nucleatum↓ IL-21/22/31↓CD40L ↓p-STAT3↓, p-STAT5 ↓ p-ERK1/2↓	Multiple intestinal neoplasia mice	Yu et al. [66]
